# Citrate of Sodium in Infant Feeding.
1Dr. F. J. Poynton, Lancet, August 13, 1904.


**Published:** 1904-09-10

**Authors:** 


### Hospital Clinics.

### CITRATE OF SODIUM IN INFANT FEEDING.[1]

In the artificial feeding of infants the desire is—a desire which has not yet been fully realised—to obtain a counterpart to human milk. In the attempts to obtain this some place stress upon the subtle composition of fresh milk, others upon its quantitative composition. The first of these schools looks to asses' or cows' milk—diluted if necessary—as a substitute for human milk, whilst the latter, insisting mainly on the percentage composition of the food, favours substituted and humanised milk. It is true that cows' milk when diluted, preferably with good drinking water that has been boiled rather than with barley water or lime water, can be converted into a food which in percentage values closely corresponds to human milk, if at the same time small quantities of cream and sugar are added. But there remains the practical difficulty of the relative indigestibility of cows' milk. In some infants this reaches an extreme degree. No dilution will so modify the curd as to render it easy of digestion in these cases. It is this personal idiosyncrasy which makes impossible the definition of a plan suitable for all cases. Again, in diluting the milk to make it more easily digested there is the risk of reducing its nutritive value below what is required for the nourishment and growth of the infant. Humanised milks endeavour to meet the difficulty by retaining the fat and reducing the proteid; but this does not modify the indigestible quality of the curd, and such milks, if sterilised for long periods, have a lowered nutritive value. Substituted milks are expensive, and often children fail to thrive on them. Patent foods, in their endless variety, are sometimes aids to digestion, and in infants over six months of age, are often useful additions to milk, but they can never be effective substitutes for milk; to rely upon them in any considerable measure is to court the development of scurvy. Neither can condensed milks satisfactorily replace fresh milk, though in hot weather they are often valuable in hospital practice. In short, except for those who can afford wet nursing or asses' milk, there is no method for the artificial feeding of an infant which is entirely satisfactory. What is wanted for the poorer classes is a method which is cheap, simple, easily applied, keeps the child more or less under medical direction, and avoids the danger of under-feeding. The addition of sodium citrate to cows' milk gives some help in these directions. The idea owes its origin to Dr. A. E. Wright, who has pointed out that there are two forms of milk-curdling. The one, due to rennet, takes place when the stomach is empty and produces a firm clot; the other is caused by acid, and the result is a loose clot. The suggestion is that much of the indigestibility of cows' milk depends upon the formation of the rennet curd. Hence, if the formation of this in the stomach can be delayed there will be an advantage, as the later formed curd is less firm and more digestible. Now it is known experimentally that if the lime salts are precipitated from milk, clotting with rennet is prevented. The problem therefore is to devise some simple method which will get rid of some part of the lime salts (and as these are in considerable excess from a nutritive point of view they can well be spared) in the hope that the delay in coagulation thus caused will result in the production of a lighter and more digestible curd. It is found in practice that this can be accomplished by the addition of sodium citrate, and a good working formula is 1 grain of the citrate to each ounce of milk, though a more accurate method is to test the milk daily with rennet and dilute acid, and to order more or less of the sodium salt according to the dense or open character of the clot. A useful procedure in busy practice is to order a solution of sodium citrate, 1 gr. to the drachm, sufficient to last for a week, adding a little chloroform water to prevent fungus formation. A teaspoonful of this solution can then be added to each feed of two tablespoonfuls of milk and the same quantity of water, say for a child four months old. If the child does not gain in weight and there is still evidence that the curd is not digested, the strength of the citrate solution can be increased. And as the proportion of milk in each feed is increased, there must also be a larger amount of the citrate used, until, when two parts of milk are being taken with one part of water, and the condition of the child is satisfactory, the use of the sodium salt may be gradually discontinued. In what may be called mild cases of milk dyspepsia the plan thus outlined has been pursued with marked success by Dr. Poynton at the Children's Hospital. It does not invariably succeed, and of course there are causes of indigestion in children other than failure to digest rennet curd, and these must not be forgotten. But in a very large number of infants which are losing ground when fed with diluted milk, etc., the sodium citrate plan works admirably. It is economical, is easily applied and adapted to varying conditions, and, apart from results, gains the confidence of the mothers who wish medicine in some form or other to be prescribed. The only drawback is some slight tendency to constipation which is easily overcome by the use of a little magnesium carbonate. But it will not cure indigestion due to organic disease, as congenital hypertrophic stenosis of the pylorus, nor will it overcome severe gastro-enteritis due to impure milk. And there are some infants who show complete intolerance of cows' milk in any form, and who cannot digest it whether treated with sodium citrate or any other addition. In these latter cases a success may often be obtained by the use of a mixture of veal-tea, whey and cream as recommended by Dr. Cheadle.

[1] Dr. F. J. Poynton, Lancet, August 13, 1904.

